# Pd-Ag Membrane Coupled to a Two-Zone Fluidized Bed Reactor (TZFBR) for Propane Dehydrogenation on a Pt-Sn/MgAl_2_O_4_ Catalyst

**DOI:** 10.3390/membranes3020069

**Published:** 2013-05-14

**Authors:** José-Antonio Medrano, Ignacio Julián, Javier Herguido, Miguel Menéndez

**Affiliations:** Catalysis, Molecular Separations and Reactor Engineering Group (CREG), Aragón Institute for Engineering Research (I3A), University of Zaragoza, Zaragoza 50018, Spain; E-Mails: 550765@unizar.es (J.-A.M.); ijulian@unizar.es (I.J.); jhergui@unizar.es (J.H.)

**Keywords:** catalytic propane dehydrogenation, membrane reactor, Two Zone Fluidized Bed Reactor, Pd-Ag membrane

## Abstract

Several reactor configurations have been tested for catalytic propane dehydrogenation employing Pt-Sn/MgAl_2_O_4_ as a catalyst. Pd-Ag alloy membranes coupled to the multifunctional Two-Zone Fluidized Bed Reactor (TZFBR) provide an improvement in propane conversion by hydrogen removal from the reaction bed through the inorganic membrane in addition to *in situ* catalyst regeneration. Twofold process intensification is thereby achieved when compared to the use of traditional fluidized bed reactors (FBR), where coke formation and thermodynamic equilibrium represent important process limitations. Experiments were carried out at 500–575 °C and with catalyst mass to molar flow of fed propane ratios between 15.1 and 35.2 g min mmol^−1^, employing three different reactor configurations: FBR, TZFBR and TZFBR + Membrane (TZFBR + MB). The results in the FBR showed catalyst deactivation, which was faster at high temperatures. In contrast, by employing the TZFBR with the optimum regenerative agent flow (diluted oxygen), the process activity was sustained throughout the time on stream. The TZFBR + MB showed promising results in catalytic propane dehydrogenation, displacing the reaction towards higher propylene production and giving the best results among the different reactor configurations studied. Furthermore, the results obtained in this study were better than those reported on conventional reactors.

## 1. Introduction

Nowadays, propylene is considered one of the most important basic products, especially in the plastics industry for polypropylene production. Propylene may be employed as a raw material for other interesting products, such as acrylonitrile, propylene oxide, different alcohols, cumene and acrylic acid. There are two main ways to produce propylene: as a byproduct in ethylene production and from off-gases in fluid catalytic cracking (FCC). Nevertheless, propylene consumption is increasing faster than ethylene consumption [1]. In addition, the increasing availability of natural gas, obtained by fracking, is reducing the use of naphtha as feedstock for ethylene production and this decreases the amount of propylene obtained by this process. Therefore, the development of on-purpose propylene production technologies is of considerable interest. Current alternatives comprise metathesis of ethylene and butenes and alkanes dehydrogenation [2]. Catalytic propane dehydrogenation (PDH) represents one of the most promising on-purpose technologies. In the PDH process, propane can be directly transformed into propylene in the presence of a selective catalyst. 

Propane dehydrogenation [Equation (1)] is a reaction limited by the thermodynamic equilibrium which main products are propylene and hydrogen. Hydrogen, as byproduct, is one of the most important energy vectors for the future [3]. Catalytic PDH is an endothermic reaction (∆H_298K_ = −124 kJ/mol) which is normally carried out at 500–600 °C and at atmospheric pressure, employing catalysts based on Pt [4,5] or Cr [6,7]. Under these operational conditions, undesirable secondary reactions take place [8,9], e.g., thermal cracking, propane hydrogenolysis or coke formation [Equations (2–4)]. Secondary reactions affect the selectivity to propylene and coke formation produces a carbon-thin layer over the catalyst active surface causing its deactivation [10,11]. As a result, the catalyst needs to be regenerated by removing the coke from its surface. Moreover, the extension of these secondary reactions increases at higher reaction temperatures, which implies an important drawback for this endothermic reaction.

C_3_H_8_ → C_3_H_6_ + H_2_ (1)

C_3_H_8_ → CH_4_ + C_2_H_4_ (2)

C_3_H_8_ + H_2_ → CH_4_ + C_2_H_6_ (3)

C_3_H_8_ ↔ 3CH_0.5_ (coke) + 3.25H_2_ (4)

Traditionally, industrial processes solve PDH limitations by employing serial reactors where reaction, purge and catalyst regeneration take place cyclically in a semi-continuous process. Other technologies co-feed propane and hydrogen mixtures in order to reduce coke deposition. As a result, lower yields to propylene are obtained.

With the aim of improving the yield to propylene and reducing the catalyst deactivation by coke deposition over the catalyst surface, several authors have proposed catalysts based on Pt alloyed with other elements such as Sn, Ga, or In [5,12,13]. Some authors have also proposed the use of Ce, K or Ni as catalyst promoters [10,14,15]. Zeolites have also been proposed as catalyst support [15,16]. In previous works by our group, Pt-Sn-K/γ-Al_2_O_3_ and Pt-Sn/MgAl_2_O_4_ catalysts were used to carry out PDH [17,18]. Pt-Sn-K/γ-Al_2_O_3_ showed higher coke formation than Pt-Sn catalyst supported on MgAl_2_O_4_ spinel, probably because of the low acidity of the latter support. The presence of Pt provided high catalytic activity towards olefins. Additionally, Sn as catalyst stabilizer improved the dehydrogenation-to-cracking ratio and reduced Pt sintering.

**Figure 1:**
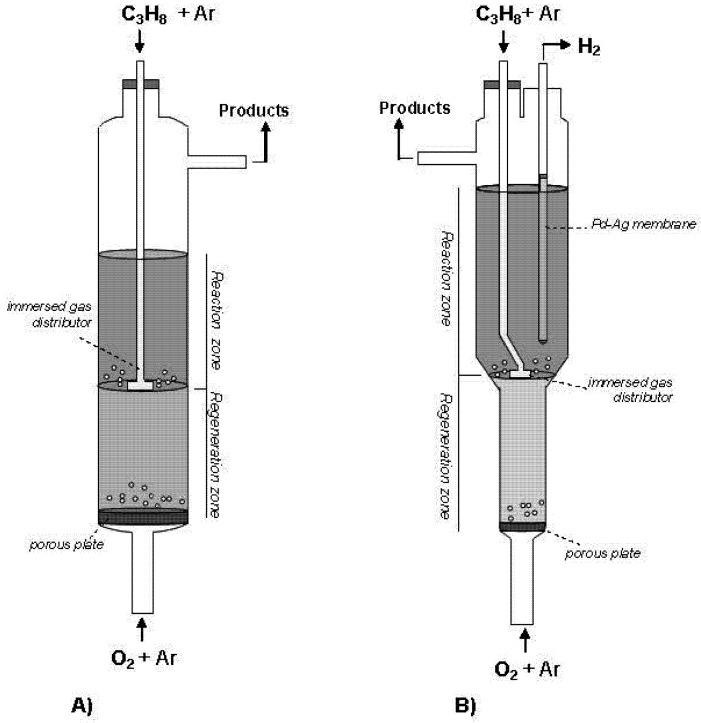
(**A**) Schematic drawing of the Two-Zone Fluidized Bed Reactor (TZFBR); and (**B**) of the Two-Section Two-Zone Fluidized Bed Reactor (TS-TZFBR) with a Pd-Ag membrane.

The TZFBR (Figure 1A) is an original reactor able to solve the limitation represented by coke formation in heterogeneous catalytic reactions [19]. This reactor has two different feed points placed at the bottom and at an intermediate point of the catalytic bed. This results in a system with two different atmospheres, *i.e.*, oxidative and reductive zones. The reactive agent (e.g., propane) is supplied in an intermediate position of the reactor. Therefore, the reaction of interest occurs in the upper zone of the fluidized bed or “reaction zone”. An oxidant agent (e.g., oxygen diluted in an inert gas) is supplied through the feed point at the bottom of the bed. This oxidant removes by combustion the coke deposited over the catalyst coming from the upper bed zone. The catalytic surface, thus, recovers its initial activity in the lower part of the reactor or “regeneration zone”. The catalyst circulation characteristic of fluidized reactors [20] implies a continuous transference of catalytic solid between the reactive and regenerative bed zones. This process integration enables catalyst activity to be maintained throughout the time on stream. By extension, the TZFBR is here proposed as a compact multifunctional solution for carrying out this kind of catalytic reactions where the catalyst suffers from a rapid deactivation. The TZFBR system has already been tested in several catalytic dehydrogenations such as catalytic propane and butane dehydrogenation with different catalysts, as well as in other applications [8,21,22]. However, an adequate optimization of the TZFBR operational conditions is required in order to achieve a suitable reactor performance [17]. The oxidizing agent should not reach the “reaction zone” because in such an event it would interact with the reactive agent, resulting in an important selectivity loss. It is also important to avoid the oxidant flow supplied at the bottom of the reactor from being too low because in such event the catalyst would not be completely regenerated.

In this work, the use of an immersed Pd alloyed membrane coupled to a TZFBR represents two-fold process intensification. For this reason, operational conditions need to be even more carefully selected in order to get a proper performance of this novel membrane reactor concept. 

Membrane reactors involve a multifunctional system, which is able to integrate a chemical reaction (e.g., oxidative reforming, hydrocarbon dehydrogenation, *etc.*) and a separation process through the membrane in the same unit [23,24]. Thus, membranes are not only considered as a separation system. Besides, they also influence the chemical reaction. The limitations of these reactors are related to the adaptability between the membrane and the reaction [25]. Typical membrane reactors include Catalytic Membrane Reactors (CMR), Packed Bed Membrane Reactors (PBMR) and Fluidized Bed Membrane Reactors (FBMR), as described in recent reviews [26,27]. 

These reactors are often employed in reactions limited by the equilibrium conversion. Membrane reactors have two main applications: for product selective separation (extractive application) or reactive supply (distributive application) [25]. The most extensively studied processes within membrane reactor technology are based on inorganic Pd and Pd alloy membranes for selective H_2_ removal in alkane dehydrogenations and catalytic reforming processes [28,29]. These processes are limited by the thermodynamic equilibrium, where a selective H_2_ removal (H_2_ is a product of the main reaction) implies an improvement in the yield of the process towards the product of interest. A limitation for the industrial implementation of these reactors is the considerable increase in coke deposition over the metal surface in reactions with hydrocarbons at high temperatures, such as dehydrogenation and reforming processes [30]. This deposition inhibits the H_2_ separation through the membrane and causes an important decrease in the process conversion [18,31].

As a multi-purpose reactor, the TZFBR coupled with a Pd or Pd alloyed membrane reactor represents an appropriate system to overcome these limitations in a PDH process. Figure 1B shows a schematic drawing of the TZFBR with a Pd-Ag membrane (TZFBR + MB). This multifunctional reactor provides *in situ* catalyst regeneration due to the TZFBR configuration, and a displacement of the main reaction towards propylene production by the selective removal of H_2_ in the “reaction zone”, *i.e.*, molecular separation through the membrane. In the present work, a novel Two-Section configuration of the TZFBR has been employed (TS-TZFBR). In order to get a better control of the reactor fluid dynamics in each reactor zone, different bed sections between both zones have been implemented to provide low regenerative-to-reactive flows if required [18,32]. The tapered bed angle between zones was selected according to the recommendations from Julián *et al.* [20], in order to avoid defluidized catalytic regions within the upper bed section. 

In a previous work, catalytic propane dehydrogenation was tested in the TS-TZFBR configuration with a hollow fiber palladium membrane employing Pt-Sn/MgAl_2_O_4 _as catalyst [17]. However, due to the limitations of hollow fiber Pd membranes, promising results were only obtained at low reaction temperatures. In the present study a commercial dense Pd-Ag membrane supported on porous stainless steel (*REB Research^®^*) is coupled to the TZFBR with the aim of testing catalytic propane dehydrogenation at higher reaction temperatures. The ability to work at higher temperatures has several process benefits such as increased propane conversion, and higher hydrogen permeation. Pd membranes supported on porous stainless steel have better mechanical resistance than Pd membranes supported on ceramic hollow fibers. Commercial tubular membranes used in this work have the following dimensions: 31.7 mm external diameter, 178 mm length and 76 µm thick Pd-Ag layer. 

The effect of the different process intensifications for the catalytic PDH on Pt-Sn/MgAl_2_O_4_ will be presented step by step, using different reactor configurations: TZFBR without oxygen (acting as a traditional Fluidized Bed Reactor, FBR), TZFBR, and multifunctional TZFBR + MB. The purpose of the experimental series with the FBR at different reaction temperatures is to evaluate the catalyst deactivation by coke deposition. The decrease in conversion with time-on-stream is related to the amount of coke deposited over the catalyst. The goal of using a TS-TZFBR for PDH is to evaluate the reactor performance in terms of *in situ* catalyst regeneration and system stability. Finally, the multifunctional TZFBR + MB is employed to illustrate the improvement in the yield to propylene by displacement of the reaction equilibrium towards products resulting from the selective hydrogen removal. This last study also deals with the PDH system stability along the time on stream and the ability of counteracting the catalyst deactivation. 

## 2. Experimental Section

### 2.1. Catalyst Preparation

Pt-Sn/MgAl_2_O_4_ was prepared in two steps. Firstly, the catalyst support was synthesized by a sol-gel method. Secondly, catalytic active compounds (Pd and Sn) were deposited on the support by wet incipient impregnation.

Magnesium (Mg(NO_3_)_2_·6H_2_O, Aldrich, 99%) and aluminum (Al(NO_3_)_3_·9H_2_O, Aldrich, 99%) compounds were dissolved and reacted for 1 h at 50 °C and pH 9 while maintaining vigorous stirring. The resulting MgAl_2_O_4_ white gel was precipitated and aged at atmospheric conditions overnight. The gel was then filtered and dried at 120 °C overnight. Finally, the support was calcined in air in two consecutive steps: first, heating the gel at 350 °C for 2 h and then at 800 °C for 8 h (2 °C/min heating rate in both steps). The MgAl_2_O_4_ support was then sieved to 75–150 µm particle size and characterized by X-ray diffraction and BET specific surface area.

The second part of the catalyst preparation consisted of coating the support with the catalytic precursors. Tin and platinum precursors (SnCl_2_, Aldrich, 98% and H_2_PtCl_6_, Aldrich, 8% dissolution in water) were deposited over the surface of the support by a wet incipient impregnation method in two consecutive steps. First, tin was impregnated during 6 h and dried at 120 °C overnight. Later, platinum was impregnated by the same method. Finally, the catalyst was calcined in air at 650 °C for 3 h (2 °C/min heating rate) and again sieved to 75–150 µm particle size.

With the aim of establishing suitable experimental conditions in the different fluidized reactors, the minimum fluidization velocity, *u*_mf_, of the catalyst was measured. This test was carried out at 550 °C in a straight FBR with Ar, the inert gas employed during the present study, and a value of *u*_mf_ = 0.53 cm/s (*i.e.*, 0.163 cm^3^(STP) cm^−2^ s^−1^) was obtained.

Before carrying out the experimental study, the catalyst had to be stabilized to guarantee the reproducibility of the results. The catalyst stabilization consisted of three cyclical steps where the catalyst was first reduced with diluted H_2_ during 2 h; then PDH was carried out in the presence of diluted C_3_H_8_ (2 h); and finally, coke deposited over the catalyst surface was removed with a stream of diluted oxygen. This cyclical process was repeated until two consecutive results were identical. The catalyst stabilization was carried out at 550 °C in a traditional fluidized bed reactor, where the conditions of the different streams in each step are summarized in Table 1.

**Table 1:** Experimental conditions during the stabilization cycles.

Variable	Reduction	Reaction	Regeneration
Feed composition	Ar:H_2_ = 3:1	Ar:C_3_H_8_ = 1:1	Ar:O_2_ = 20:1
*u*_r_	3	3	3
Time (h)	2	2	Until CO and CO_2_ signals not seen
*W*_0_/*F*_C3H8_ (g min mmol^−1^)	–	12.2	–

### 2.2. Materials Characterization

X-Ray diffraction data were obtained at 298 K in a “D-Max Rigaku” diffractometer with mobile anode. This works at 40 kV and 80 mA with a Cu anode and graphite monochromator to select a CuKα1.2 radiation. The diffraction angle during the measurements was varied between 10° and 90° with a 0.03° step. The BET specific surface of the catalyst was obtained in a “Micrometrics ASAP 2020”.

Commercial Pd-Ag membranes were characterized by the permeation flux at different temperatures and pressures. These tests were carried out with the membrane inside the reactor, but without the presence of the catalytic bed. For this purpose, a constant flow rate of H_2_ + Ar mixture was supplied at the bottom inlet of the reactor. When a pressure drop across the membrane was applied with a vacuum pump, hydrogen permeated selectively through the membrane. This resulted a reduced outlet gas flow in the reactor related to the removal of hydrogen through the membrane. Hydrogen permeation results were, therefore, obtained by difference between retentate side outlet flows with and without applying certain pressure drop. Membrane permeation studies were carried out employing different H_2_/Ar ratios and temperatures. Finally, a closure test with pure Ar was carried out to verify the selectivity of the Pd alloy membrane. The retentate outflow measured in this test was the same with and without vacuum. This indicated that no Ar was flowing through the membrane, illustrating the membrane full selectivity towards hydrogen.

### 2.3. Reaction

Catalytic propane dehydrogenation was carried out in two different reactor facilities. A conventional FBR was first employed during the catalyst stabilization step. This reactor consisted of a 2.8 cm inner diameter quartz tube where the gases are fed at the bottom inlet of the reactor. The TZFBR + MB (Figure 1B) consisted of a quartz column with different sections in the reaction and the regeneration zones. The upper and lower inner diameters of the reactor were 3.0 and 1.8 cm, respectively, with a 60° angle (with respect to the horizontal position) in the transition between both zones. Having a different section in the lower zone provides better control of the fluid dynamic regime in both regions compared to a straight column reactor [31]. The oxidant agent (diluted oxygen) is fed through the bottom of a 40 µm porous quartz plate, which acts as gas distributor and catalytic bed support inside the reactor. Propane is fed at an intermediate point of the transition bed section. This reactive gas is fed through a 4-axis immersed gas distributor with 4 mm external diameter. The commercial Pd-Ag membrane was placed inside the reaction zone. The membrane consists of a dense layer of 1/8′′ (3 mm) external diameter (0.03′′ wall) and 5′′ (12.7 cm) in length, sealed at one end and brazed to a stainless steel stub at the other. Its high mechanical resistance means that it can be immersed directly in the fluidized bed without an external membrane-shell for its protection, which was not the case in our previous works with Pd-membranes [17,18]. Both the commercial membrane and the propane distributor were connected to the top wall of the reactor.

Each reactive gas flow was controlled by a set of mass flow controllers. The furnace temperature control was carried out by an immersed thermocouple (Controller 3116, Eurotherm). Reaction products were analyzed by online gas chromatography (µGC-R3000, SRA Instruments) and a vacuum pump (2P-3, Start) was employed to generate the vacuum inside the membrane for the hydrogen permeation. 

A heating rate of 2 °C/min was employed to establish the reaction temperature, with the aim of avoiding membrane damage. Before the reaction started, the catalyst was activated for 2 h at 550 °C with hydrogen to reduce the platinum oxide species to Pt^0^. Catalytic propane dehydrogenation was, then, carried out at different reaction temperatures between 500° and 575 °C and with different *W*_0_/*F*_C3H8_ ratios (*i.e.*, ratio of catalyst mass to propane molar flow) between 15 and 35 g min mmol^−1^, under the conditions described in the Table 2. A catalyst mass of 70 g was employed during the entire experimental series. After each experiment, the catalyst was fully regenerated with a diluted oxygen stream at 550 °C until CO and CO_2_ signals were not detected by gas chromatography.

**Table 2:** Experimental series.

Variable	FBR (TZFBR without oxygen)	TZFBR	TZFBR + MB
*T* (°C)	500–575	500–575	500–575
*u*_r,regen_ (–)	2.5	2.5	2.5
*u*_r,react_ (–)	1.75	1.75	1.75
Oxygen (%)	–	1–5	1–5
*W*_0_/*F*_C3H8_ (g min mmol^−1^)	21.1	15.1–35.2	15.1–35.2
∆ *P* (bar)	–	–	1.1

## 3. Results

**Figure 2:**
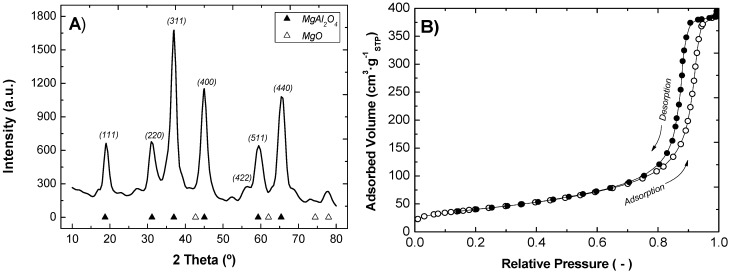
(**A**) XRD pattern; and (**B**) N_2_ adsorption-desorption isotherms at 77 K for MgAl_2_O_4_ support.

Figure 2A shows that spinel MgAl_2_O_4_ was properly synthesized since the peaks in the X-Ray diffraction agree with its theoretical structural geometry. The adsorption isotherm represented in Figure 2B shows a typical type-4 adsorption curve, which is the characteristic isotherm of mesoporous solids. This is corroborated by the BJH analysis, where the average pore size obtained was 15.2 nm. The BET area shows high values (146 m^2^ g^−1^) with a suitable pore volume (0.61 cm^3^ g^−1^).

**Figure 3:**
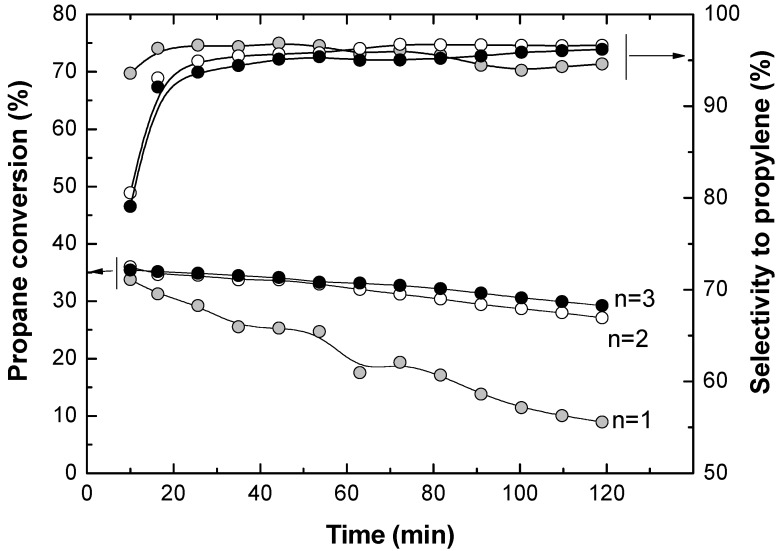
Propane conversion and selectivity to propylene for three stabilization (reduction + reaction + regeneration) cycles of the fresh Pt-Sn/MgAl_2_O_4_ catalyst.

Consecutive stabilization cycles (reduction-reaction-regeneration) were carried out at 550 °C in a traditional fluidized bed reactor (FBR) until two consecutive cycles with similar results were obtained. The objective was to ensure PDH process reproducibility. Figure 3 shows curves of propane conversion (*X*_C3H8_) and selectivity to propylene (*S*_C3H6_) at different cycles, where the differences in performance between the first and the consecutive cycles are related to the catalyst stabilization process. It can be observed that results from the second and the third cycle were similar, indicating that a stable catalyst performance had been reached. 

With the aim of studying the influence of the reaction temperature on the catalyst deactivation, different experiments were carried out in the TZFBR without feeding oxygen in the lower zone. The reactor performance was similar to that of a traditional fluidized bed reactor. With this reactor configuration, the final comparison between all configurations can be made more easily because the physical reactor is the same for all experiments. 

**Figure 4:**
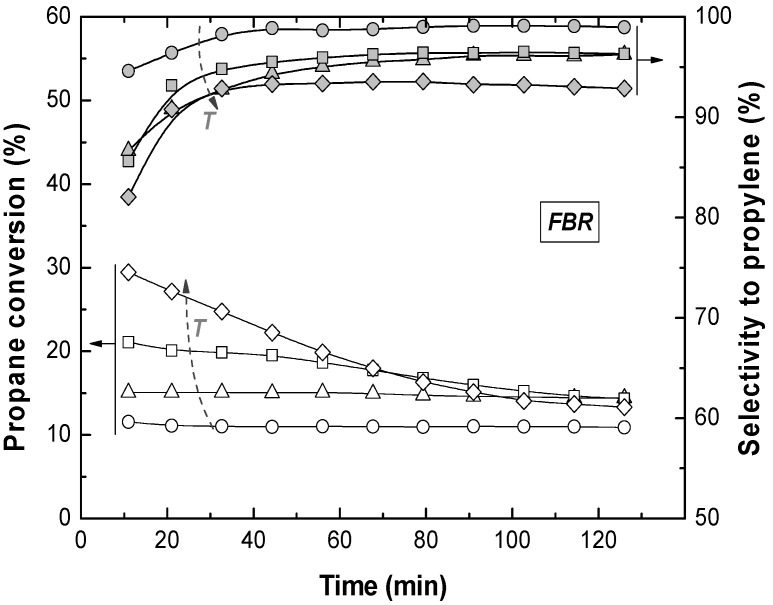
Propane conversion and selectivity to propylene for three stabilization (reduction + reaction + regeneration) cycles of the fresh Pt-Sn/MgAl_2_O_4_ catalyst.

The results for this experimental series in the TZFBR without oxygen being fed in the lower zone are shown in Figure 4. It can be seen that a high reaction temperature favors higher propane conversion, but lower selectivity to propylene. This is due to the secondary reactions such as cracking [Equations (2) and (3)] and coking [Equation (4)], which are favored at high temperatures. These results suggest that carrying out catalytic propane dehydrogenation in a traditional fluidized bed reactor is not efficient. The catalyst deactivation by coke deposition requires the use of a second reactor where the catalyst can be regenerated. 

**Figure 5:**
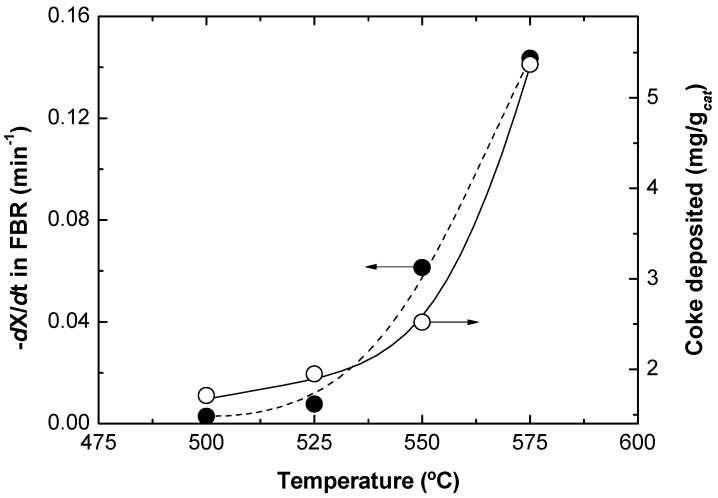
Propane conversion decay in the TZFBR without oxygen (measured as −d*X*_C3H8_/d*t*), and amount of coke formed during reaction, both as a function of the temperature. Lines present as a visual help only.

Figure 4 shows that catalyst deactivation was higher when the reaction temperature was increased. It was found that the average slope of the propane conversion along the time on stream was larger at higher temperatures. This means that coke was deposited faster at high temperatures, implying a quicker catalyst activity loss. This catalyst activity loss has been measured at each temperature as the slope (with the sign changed) in the linear regression of the experimental data (−d*X*/d*t*). This slope at each reaction temperature is represented in Figure 5. The amount of coke deposited over the catalyst was determined during the regeneration step. Results show that the higher the reaction temperature, the higher the amount of coke deposited, as can also be seen in Figure 5. This confirms that the catalyst deactivation rate and the amount of coke formed in the reaction are linearly related.

**Figure 6:**
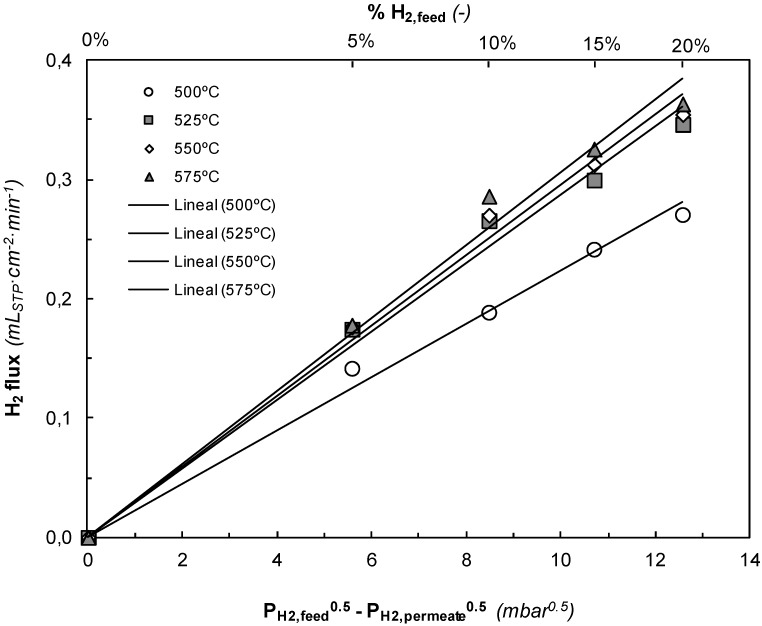
H_2_ permeating flux *vs.* Sieverts’ driving force for Pd-Ag membrane at different temperatures (550–575 °C), *Q*_gas_ = 200 mL_STP_ min^−1^, *P*_permeate_ = 1 mbar.

After proceeding with the experimental series in the multifunctional reactor, the results of the membrane characterization are shown in Figure 6. 

Inert gas did not permeate through the Pd-Ag layer under the conditions studied with a gas stream of pure Ar. This implies that the membrane consists of a defect-free continuous alloy layer and infinite H_2_ perm-selectivity (S_H2/Ar_ = ∞). Permeation tests as those shown in Figure 6 were carried out before and after a number of experimental series in PDH reactive conditions, showing similar hydrogen permeation and selectivity. This result confirms good membrane stability. The almost linear H_2_ permeation trend against the square root of the pressure drop indicates that H_2_ permeation takes place via metallic diffusion, where the permeating flux is a function of the Sieverts’ driving force. Moreover, the permeation flux is also affected by the temperature. Hydrogen permeation increases linearly with the difference of the square root of the H_2_ partial pressure at both sides of the membrane, which implies that the permeation is controlled by proton diffusion through the Pd alloy membrane. Besides, higher temperatures provide higher hydrogen fluxes because the diffusion is favored.

**Figure 7:**
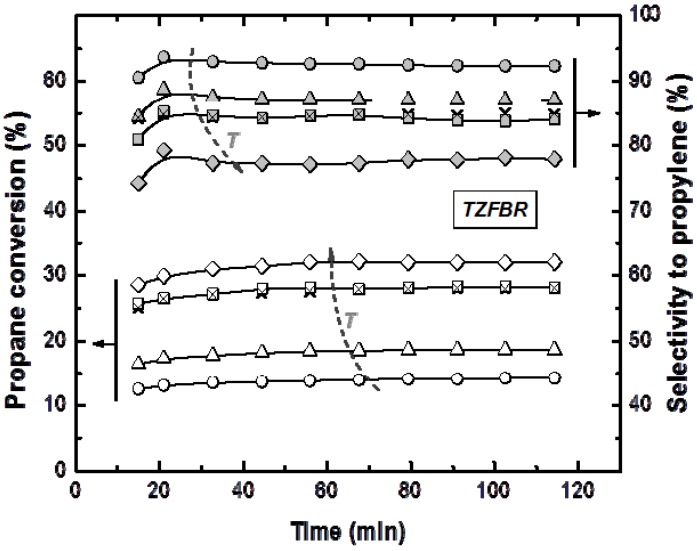
Propane conversion and propylene selectivity at different reaction temperatures (○ 500 °C; ∆ 525 °C; □ and × 550 °C; ◊ 575 °C) in the TZFBR, working at the optimum oxygen percentage. Operating conditions are similar to Figure 4.

**Figure 8:**
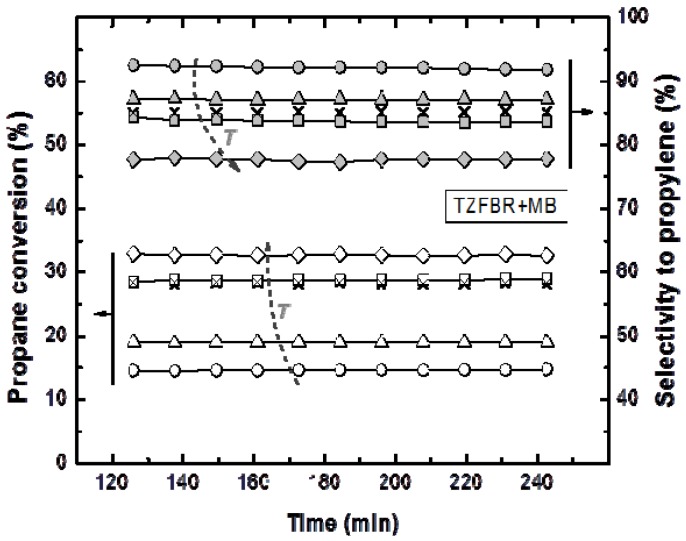
Propane conversion and propylene selectivity at different reaction temperatures (○ 500 °C; ∆ 525 °C; □ and × 550 °C; ◊ 575 °C) in the Membrane (TZFBR + MB), working at the optimum oxygen percentage. Other operating conditions are similar to Figure 4.

Reactions in the TZFBR and in the TZFBR + MB were carried out consecutively, without catalyst regeneration in between. Firstly, the TZFBR + MB unit was used as a conventional TZFBR in which no pressure drop was applied across the Pd-Ag membrane. Once PDH achieved a steady state in this reactor configuration, the vacuum pump was connected. The system behaved then as a membrane reactor and hydrogen could be removed. As a result, the effect of the Pd-Ag membrane on the steady state operation of the conventional TZFBR could be analyzed in a single experiment and reactor unit by just switching on/off the vacuum pump. Figure 7 shows the evolution of propane conversion and selectivity to propylene at several reaction temperatures for the first 2 h, when the system operated as TZFBR. The system reached a steady state catalytic activity at all reaction temperatures due to the *in situ* catalyst regeneration. Oxygen requirements at each temperature correspond with an optimum value, enough to counteract the catalyst deactivation but not so large that oxygen can reach the reaction zone. This optimum was determined in a previous work [17] for each temperature, being roughly 1%, 2%, 3%, and 5% (expressed as the percentage of oxygen fed in the regeneration zone relative to the total gas fed to the reactor) for 500, 525, 550, and 575 °C, respectively. After 2 h of reaction, the vacuum pump was turned on and hydrogen was selectively removed from the reaction zone through the Pd-Ag membrane, modifying the conversion reached. The new equilibrium was reached faster than in the previous configuration, obtaining constant values for selectivity to propylene and propane conversion from the first analysis (Figure 8). This was because the new stationary state was close to the previous one attained in the TZFBR configuration. An improvement in the yield to propylene was observed at all the reaction temperatures. Furthermore, the yield to propylene remained constant because of the performance of the multifunctional reactor, where catalyst is regenerated in the lower zone. This represents a substantial improvement of the system compared to membrane reactors, which provide higher coke formation in dehydrogenation reactions ending up with catalyst deactivation and a significant decrease in the process yield. In all cases the TZFBR + MB has shown an improvement in the results in relation to the TZFBR, with ∆*Y*_C3H6_ = 2.54%, 1.97%, 1.88%, and 1.83% at 500, 525, 550, and 575 °C, respectively. Better relative improvements were obtained at low temperatures. This implies that the increase in the propane dehydrogenation rate by raising the temperature is greater than the increase in the hydrogen permeation flux through the membrane by the same factor. 

**Figure 9:**
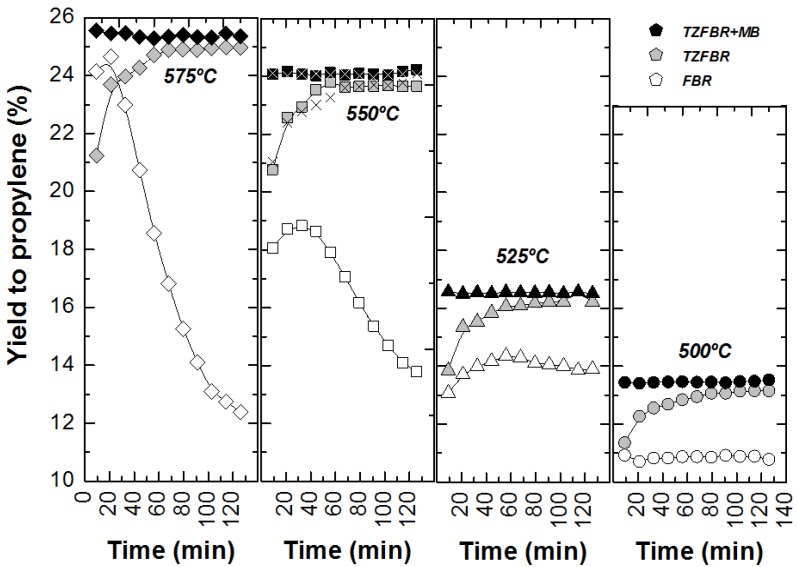
Yield to propylene with different reactor configurations and temperatures. Operating conditions as in Figure 4 (FBR), Figure 7 (TZFBR), and Figure 8 (TZFBR + MB).

Comparative results between the three different reactor configurations at different reaction temperatures are shown in Figure 9. In all cases, the TZFBR + MB was found to be the best reactor configuration for catalytic PDH, followed by the TZFBR configuration. Both enabled the catalyst activity to be maintained during the reaction time, due to the multifunctional performance of the reactor. In contrast, the TZFBR working without oxidant (a configuration similar to a traditional FBR) showed the worst results, suffering catalyst deactivation at all reaction temperatures.

In order to assess the reproducibility of the results in the TZFBR and TZFBR + MB configurations, several repeated but not consecutive experiments were carried out under identical operating conditions. As an example, for the repeated run at 550 °C, conversion, selectivity, and yield to propylene are presented in Figure 7, Figure 8, Figure 9 (× symbols), showing excellent agreement with those previously obtained at that temperature.

**Figure 10:**
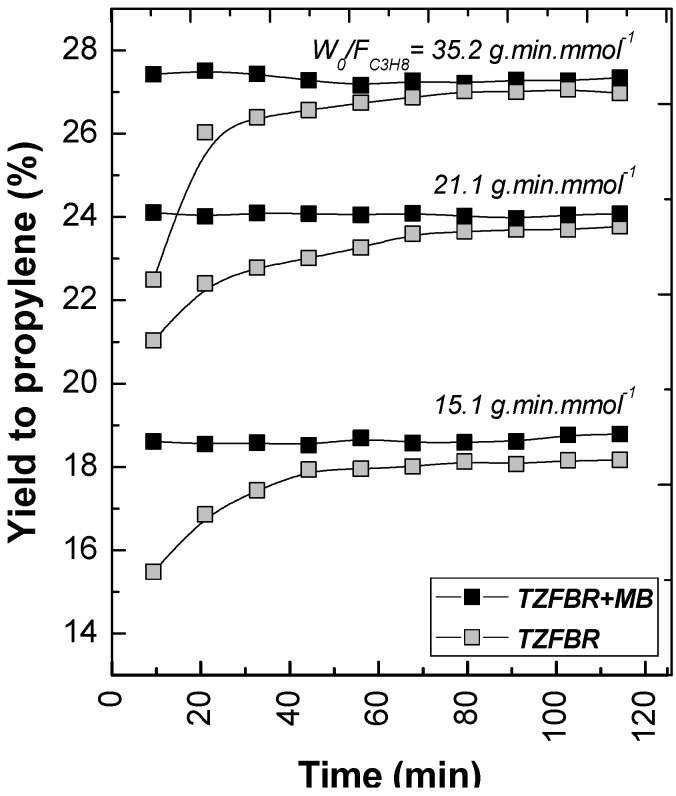
Yield to propylene with different *W*_0_/*F*_C3H8_ ratios for TZFBR and TZFBR + MB configurations. *T* = 550 °C; *W*_0_ = 70 g; *u*_r,regen_ = 1.75; *u*_r,react_ = 2.5; O_2_ percentage = 3%; C_3_H_8_ percentage = 30% (*W*_0_/*F*_C3H8_ = 15.1 g min mmol^−1^), 50% (*W*_0_/*F*_C3H8_ = 21.1 g min mmol^−1^), and 70% (*W*_0_/*F*_C3H8_ = 35.2 g min mmol^−1^).

The effect of modifying the *W*_0_/*F*_C3H8_ ratio in both the TZFBR and the TZFBR + MB configurations was analyzed by means of a series of experiments carried out at constant temperature (*T* = 550 °C) and constant catalyst load (*W*_0_ = 70 g). Different propane flow rates were used for this purpose, with different percentages of propane in the total gas fed to the reactor. As expected, the higher the residence time in the reaction zone (*i.e.*, high *W*_0_/*F*_C3H8_), the higher the conversion achieved. However, significant differences in selectivity to propylene were not obtained. As a result, the yield to propylene increased with *W*_0_/*F*_C3H8_, being always higher for the TZFBR + MB configuration as can be seen in Figure 10. In these results, a better relative improvement was observed for low *W*_0_/*F*_C3H8_ ratios. In fact, ∆*Y*_C3H6_ = 2.69%, 1.85%, and 1.12% at 15.1, 21.1, and 35.2 g min mmol^−1^, respectively. It can be assumed that lower propane conversions and higher hydrogen partial pressures were achieved by working with low *W*_0_/*F*_C3H8_ ratios (*i.e.*, high propane percentages in the total gas feed to the reactor), making the hydrogen removal effect of the membrane more relevant.

**Figure 11:**
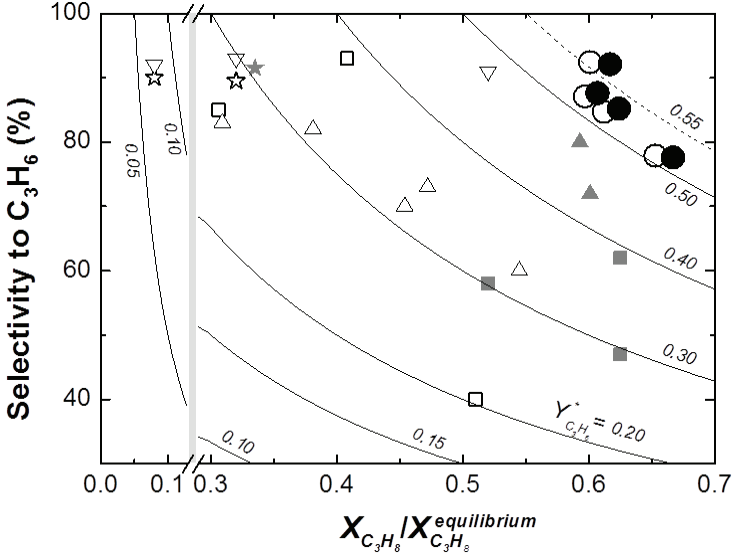
Selectivity *vs.* (conversion/equilibrium conversion) for TZFBR and TZFBR + MB runs from Figure 8 and comparison with results from the literature: 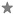 Jablonsky *et al.*, 1999 [13], 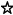 Assabumrungrat *et al.*, 2000 [33], 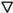 Salmones *et al.*, 2002 [34], 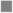 Schäfer *et al.*, 2003 [35], 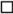 Nawaz *et al.*, 2009 [16], 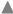 Chen *et al.*, 2010 [36], 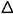 Wang *et al.*, 2012 [37], ○TZFBR (Figure 7), ● TZFBR + MB (Figure 8). Diverse operating conditions. Lines in graph represent constant values of Y*_C3H6_ (*i.e*., ratio “yield to C_3_H_6_/equilibrium conversion of C_3_H_8_”).

Finally, the results obtained in the TZFBR and TZFBR + MB multifunctional reactors are compared with previously published results for catalytic propane dehydrogenation with different reactor configurations (Figure 11) [13,16,33,34,35,36,37]. Results obtained at different operating conditions were normalized by dividing the experimental propane conversion by the equilibrium conversion in each case.

Figure 11 shows that the yield to propylene divided by the equilibrium conversion of propane in a conventional reactor (Y*_C3H6_) obtained with the TZFBR + MB was higher than with other reactor configurations published in the literature. Moreover, literature results correspond to instantaneous values during the experiments where catalyst deactivation was taking place. By contrast, the values obtained with both the TZFBR and the TZFBR + MB correspond to a steady state during the time on stream, because coke deposition over the catalyst surface was counteracted *in situ* by coke removal in the lower zone of the multifunctional reactor.

## 4. Conclusions

Different reactor configurations have been tested for catalytic propane dehydrogenation under different operational conditions employing Pt-Sn/MgAl_2_O_4_ as a catalyst. This catalyst showed good performance for the reaction studied, with a lower coking tendency than the Pt-Sn/γ-Al_2_O_3_ used in previous works. Results obtained using the FBR configuration (*i.e.*, without supplying oxygen in the lower zone of the bed) at different reaction temperatures confirmed that coke formation (and its deposition over the surface of the catalyst) causes significant catalyst deactivation and a continuous decrease in the yield to propylene during the reaction. The TZFBR is an interesting multifunctional reactor in which catalyst can be continuously regenerated due to the presence of two different atmospheres (zones) inside the catalytic bed. When the amount of oxygen fed is optimal, a steady state can be achieved at different reaction temperatures. In addition, coupling a selective Pd-Ag membrane with the TZFBR produces a novel tri-functional reactor, which enables a twofold process intensification for catalytic propane dehydrogenation. 

A commercial Pd alloyed membrane has shown high selectivity to hydrogen. The use of a membrane coupled in the reaction zone of the TZFBR has improved the equilibrium limited process by displacement of the main reaction towards the products. Furthermore, the yield to propylene achieved a constant value due to the suitable optimization of the reactor in its regeneration zone. The best results for catalytic propane dehydrogenation were obtained in the TZFBR + MB. The improvement of the membrane reactor compared to the TZFBR was better at low reaction temperatures and at low catalyst load to molar flow of fed propane ratios, according to the higher relative increase in propylene yield. A standardized comparison has shown that the multifunctional reactor provided better propylene yield than other reactor configurations reported in the literature.

Future work will focus on increasing the ratio of the permeation flux through the membrane to hydrogen produced by the reaction, with the aim of achieving a higher conversion increase. We suggest two main alternatives: to increase the membrane surface (coupling more than one membrane to the TZFBR) or to design a novel TZFBR with smaller total flows through the catalytic bed. Working with a lower total feed would mean the removal of a larger percentage of the formed hydrogen.

## Nomenclature

d*X*/d*t*: propane conversion variation (min^−1^)
d*Y*/d*t*: yield to propylene variation (min^−1^)
FBR: Fluidized Bed Reactor
molar flow of propane (mmol min^−1^)
*n*: stabilization cycle number
*P*_permeate_: pressure in the permeate side (mbar)
*Q*_gas_: volumetric flow (mL_STP_ min^−1^)
selectivity to propylene (%)
H_2_ perm-selectivity: ratio of the permeation flux of pure H_2_ to pure Ar
*T*_r_: reaction temperature (°C)
TZFBR: Two-Zone Fluidized Bed Reactor
TS-TZFBR: Two-Section Two-Zone Fluidized Bed Reactor
TZFBR + MB: Two-Zone Fluidized Bed Reactor with Membrane
*u*_mf_: minimum fluidization velocity (cm^3^ cm^−2^ s^−1^)
*u*_r,react_: relative velocity in the reaction zone (–)
*u*_r,regen_: relative velocity in the regeneration zone (–)
*u*_r_: relative velocity (–)
*W*_0_: catalyst weight (g)
propane conversion at equilibrium (%)
propane conversion (%)
yield to propylene (%)
relative yield to propylene: (–)
∆*P*: partial pressure difference across the membrane (bar)
relative increase in yield to propylene from TZFBR to TZFBR + MB configurations (%)
